# Influence of Glass Additions on Illitic Clay Ceramics

**DOI:** 10.3390/ma13030596

**Published:** 2020-01-28

**Authors:** Andrei Shishkin, Janis Baronins, Viktors Mironovs, František Lukáč, Igor Štubňa, Jurijs Ozolins

**Affiliations:** 1 Rudolfs Cimdins Riga Biomaterials Innovations and Development Centre of RTU, Institute of General Chemical Engineering, Faculty of Materials Science and Applied Chemistry, Riga Technical University, 3 Pulka Str., LV-1007 Riga, Latvia; jurijs.ozolins@rtu.lv; 2 Department of Environmental Science, Faculty of Geography and Earth Sciences, University of Latvia, LV-1004 Riga, Latvia; janis.baronins@lu.lv; 3 Scientific Laboratory of Powder Materials, Faculty of Civil Engineering, Riga Technical University, 6A Kipsalas Str., room 110, LV-1048 Riga, Latvia; viktors.mironovs@gmail.com; 4 Institute of Plasma Physics, The Czech Academy of Sciences, Za Slovankou 3, 18200 Prague 82, Czech Republic; lukac@ipp.cas.cz; 5 Department of Physics, Constantine the Philosopher University, A. Hlinku 1, 94974 Nitra, Slovakia; istubna@ukf.sk

**Keywords:** clay, illite, glass, waste glass, ceramics, specific strength, compressive strength

## Abstract

A mixture of an illitic clay and waste glass was prepared and studied during the sintering process. The illitic clay, from the Liepa deposit (Latvia), and green glass waste (GW) were disintegrated to obtain a homogeneous mixture. The addition of disintegrated GW (5–15 wt% in the mixture) led to a reduction in the intensive sintering temperature, from 900 to 860 °C, due to a significant decrease in the glass viscosity. The addition of GW slightly decreased the intensities of the endo- and exothermic reactions in the temperature range from 20 to 1000 °C due to the reduced concentration of clay minerals. GW reduced the plasticity of the clay and reduced the risk of structural breakage. The increase in sintering temperature from 700 to 1000 °C decreased the apparent porosity and water uptake capacity of the ceramics from 35% and 22%, down to 24% and 13%, respectively. The apparent porosities of all the sintered mixtures showed a decrease of between 6% to 9% after the addition of GW with concentrations from 5 up to 15 wt% respectively, while the water uptake capacities decreased from between 4% and 10%. The addition of GW led to an increase in the apparent density of the ceramic materials, up to 2.2 g/cm^3^. Furthermore, the compressive strength increased by more than two times, reaching a highest value of 240 MPa after the sintering of the 15 wt% GW-containing mixture at 1000 °C.

## 1. Introduction

Between 1995 and 2015, the overall waste generation rate in the European Union increased by 366% [1]. In 2016, about 16.29 billion tons was generated by the second most common (with 3.5–4.2% per year growth tendency) commercially available packaging material–glass [2]. Pollution risks inevitably cause demands for more intensive recycling efforts. The utilization of glass waste in clay ceramic products provides such benefits like decreased sintering temperature [3], reduced shrinkage coefficient during the drying and sintering processes [4], and reduced water absorption coefficient of the ceramics [5].

Sustainability principles require innovations for the more efficient and rational consumption of nationally available raw materials into waste-free products. The design of highly porous clay ceramics with relatively high specific surface area, reduced density [6], durability under thermal shock and chemical attack conditions, and sufficiently high mechanical strength qualifies as high-value-added widely applicable materials, which may offer considerable potential for the beneficial integration of glass waste.

The dominant particle size and its distribution characterize the essential properties of the clay feedstock. Fine-grained (so-called “fat”) clay is plastic and effectively maintains its shape during the drying process. However, relatively high drying shrinkage may cause cracking, leading to structural failure. More roughly grained (also called “stove”) clay exhibits a lower shrinkage coefficient during drying [7]. However, a decrease in particle size reportedly led to an increase in the dimensional changes during the firing of an illitic clay [8]. Besides, the dimensional stability of shaped clay becomes challenging [7,9]. The addition of finely milled glass powder commonly reduces the plasticity of wet clay; however, the effect on the porosity of the ceramic material must be considered.

In the scientific literature, there is a lot of information on different types of glass additions to clay [10]. The kaolinite-type clays’ interaction with glass has been widely studied [11,12,13]. Very often, glass-clay ceramics are described without any reference to clay type [14]. Ondruška et al. [15] described the influence of waste glass addition on the thermal properties of kaolin and illite clays, but the main parameters that characterize ceramics, such as porosity, shrinkage, and mechanical properties, are not provided in their studies. 

In Latvia, Slovakia, the United Kingdom, and other countries, illitic clays are widely distributed and used. The bottle glass cullet and illitic clay have high importance because these materials are widespread in Europe, in particular, and in the world in general. The lack of systematic studies of the illitic clay and bottle glass cullet sintering behavior and the properties of the ceramic materials provide justification for such a study. Some reports have shown the utilization of biomass in porous ceramic material for building applications [16,17,18,19], but the mechanical properties of the bricks were shown to deteriorate. On the other hand, the addition of glass cullet (GC) to the initial clay led to a strengthening of the ceramic body and better performance in terms of mechanical properties and porosity [20,21,22]. The use of packaging glass waste in the ceramic industry shows promising results as the glass addition does not lead to a severe deterioration of mechanical properties. Beneficial properties of small amounts of glass addition (up to 20 wt%) were reported by Jimenez-Millan [23].

Herein, the effect of the most common municipal waste, selected green colored bottle GC, on the properties of ceramic materials based on the clay from Liepa’s clay deposit, representing the applicability of the two-rotor-type disintegrator to produce clay-glass powder type feedstock, is reported. The present research aims to investigate the illite clay-glass system properties with 5–15 wt% waste glass addition, up to temperature levels of 1000 °C.

## 2. Materials and Methods

### 2.1. Materials

Currently, the Liepa clay deposit is the most developed Devonian clay site commercially exploited in Latvia. The chemical composition of the used clay – Liepa’s deposit (Lode LTD, Liepa, Latvia), as determined in a previous work [24], and bottle glass [25] are indicated in Table 1. Mixtures of clay-GC with the glass concentrations of 0, 5, 7, 10, 13, and 15 wt% of GC were prepared. Samples of clay-GC with the glass concentrations of 0, 5, 7, 10, 13, and 15 wt% of GC were prepared by adding 14 wt% distilled water to dry clay-GC mixtures, which were subsequently mixed to obtain a homogeneous plastic mass, which was inserted in the extruder. Cylindrical samples, with a diameter and height of 14 ± 1 and 18 ± 1 mm, respectively, were extruded. The extruded samples were dried in open air for 72 h, then in an oven, MEMMERT ULE 400 (Memmert GmbH + Co. Schwabach, Germany), for 24 h, and subsequently sintered in a furnace, Nabertherm LHT 08/17 equipped with controller P330 (Nabertherm GmbH, Lilienthal, Germany), at temperatures of 700, 800, 900, 1000, 1050, and 1100 °C in an air atmosphere, at a heating rate of 5 °K/min and a dwell time of 30 min at the highest temperature. The height of the obtained samples was sawn down to 14 ± 0.5 mm with help of the Buehler IsoMet Low Speed Saw (Illinois Tool Works, Glenview, IL, USA). Seven samples were prepared for each experimental point (GC loading and firing temperature).

### 2.2. Feedstock

Homogenized clay from Liepa’s clay deposit (Lode LTD, Liepa Latvia) was crushed by a hammer-crusher into agglomerates with sizes down to 20–30 mm, jaw-crushed down to 1–5 mm, and subsequently dried to a constant weight in the MEMMERT ULE 400 (Memmert GmbH + Co. KG, Schwabach, Germany) oven at 105 °C. Selected municipal-waste green-colored glass bottles were washed, dried, hammer-crushed into cullets with particle sizes down to 20–25 mm, and jaw-crushed down to 1–5 mm. The high-performance vibratory sieve shaker Analysette 3 (FRITSCH GmbH, Idar-Oberstein, Germany), equipped with sieves with a nominal sieve opening from 0.045 up to 4 mm, was used for granulometric analysis of the jaw-crushed materials of sample weight 100 g. Distilled water was used for the preparation of the ceramic samples.

### 2.3. Milling and Mixing by Disintegration

Disintegration provides intensive grinding of a powder with the effect of high -energy shifting during impacts. High linear speed causes particle collisions to the surface (target, grinding body) inside the disintegrator chamber, generating higher stresses than the strength of the treated material [26]. Grindable particles collide at relative velocities up to 200 m·s^−1^, which leads to a significantly increased surface area and chemical reactivity of the disintegrated product [27].

**Figure 1:**
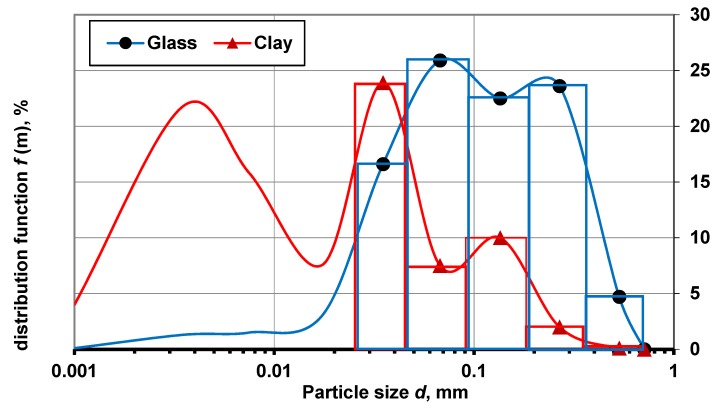
Particle size distribution of the clay and glass after the second milling/mixing.

The disintegrator DSL-175 setup (Tallinn University of Technology, Tallinn, Estonia) [28,29,30] was used for the clay and GC milling, and clay-GC dry mixture preparation. The preparation of the clay-GC dry mixture comprised three stages: (i) Separate primary milling of each component by disintegration; (ii) blending of clay and GC, in certain defined proportions, in a plastic container; (iii) additional treatment of the dry clay-GC blend by the disintegrator DSL-175 for finer milling and better mixing. For determination of the clay and glass particle distribution, parallel secondary milling, by the disintegrator, of the clay and GC was done separately. To classify the milled product, a Fritsch Analysette3Pro (FRITSCH GmbH, Idar-Oberstein, Germany) sieving machine and the following sieves were used: 5.6, 2.8, 1.4, 0.71, 0.355, 0.180, 0.090, 0.045, and 0.025 mm. The fine fraction that was left on the meshes below 0.025 mm was then weighed and used for thorough analysis in the laser analyzer Fritsch Analysette 22 (FRITSCH GmbH, Idar-Oberstein, Germany). The percentages of the fractions were calculated and the particle size distribution curves were determined. The particle size distribution after the second milling/mixing is presented in Figure 1 as a combination of a histogram (for particles from 0.025 to 1.00 mm) and a curve for particle sizes below 0.025 mm. The degree of disintegration (*n*) was calculated according to Equation (1):

(1) $$n=\frac{D_{50}}{d_{50}},$$

where

D_50_—average particle size before disintegration, mm;
*d_50_*—average particle size after disintegration, mm.

### 2.4. Characterization of the Sintering Process and Phase Transformation

Thermogravimetric (TG) and differential thermal analysis (DTA) of the samples were performed during heating from 20 °C to 1100 °C under an air atmosphere with a heating rate of 5 °K/min using the analyzer DERIVATOGRAPH Q-1000 (MOM SZERVIZ KFT., Budapest, Hungary). 

Thermodilatometric (TD) analysis was carried out using the high-temperature optical microscope, EM201 HT163 (Hesse instruments, Germany), during heating from 20 °C to 1300 °C in an air atmosphere at a heating rate of 5 °K/min.

Phase compositions of the samples were determined by the powder X-ray diffraction (XRD) method in a symmetrical Bragg–Brentano geometry on a D8 Discover (Bruker AXS, Karlsruhe, Germany) using Cu-Kα radiation and a divergent beam. Quantitative Rietveld refinement was performed in a TOPAS V5, aiming to determine the wt% of all the identified phases following the theory from [1,2].

A beam knife above the sample was used in order to suppress air scattering into the 1D X-ray detector to enable the determination of the amorphous content. Phase identification was done using the X’Pert HighScore program, which accessed the PDF-2 database of crystalline phases. 

### 2.5. Characterization of Physico-Mechanical Properties

The apparent porosity and apparent density of the sintered samples were measured using Archimedes’ principle in compliance with the standard LVS EN ISO 10545-3: 2002 [31]. Shrinkage of the samples was measured by digital calipers (resolution: 0.01 mm) before and after firing. The compressive strength of the sintered samples was measured with the mechanical tester Instron 8801 Universal Testing Machine (Instron, Darmstadt, Germany). The compression tests were performed at a constant speed of 1 mm·min^−1^ up to a deformation limit of 10%. Seven specimens were used for each experimental point (GC loading and firing temperature combination) for compression, and the Archimedes tests.

## 3. Results and Discussion

### 3.1. Efficiency of Clay and Glass Disintegration

The first disintegration of the clay resulted in *n*_average_clay_ = 260 and an increased ratio of finer particles (*d_50_*__clay_ = 0.035 and 0.0035 mm) from 30% up to 70%. Repeated disintegration of the glass led to a relatively homogeneous granulometric content with *n*_average_glass_ = 25, and d_50_glass_ decreased from 0.25 down to 0.1 mm, as shown by the combined bar-line diagram in Figure 1. The total specific electrical energy was 6.8 kW·h·t^−1^ (24.48 kJ/kg) for the double disintegration milling. 

### 3.2. Effect of the Glass Concentration on the Sintering Processes

**Figure 2:**
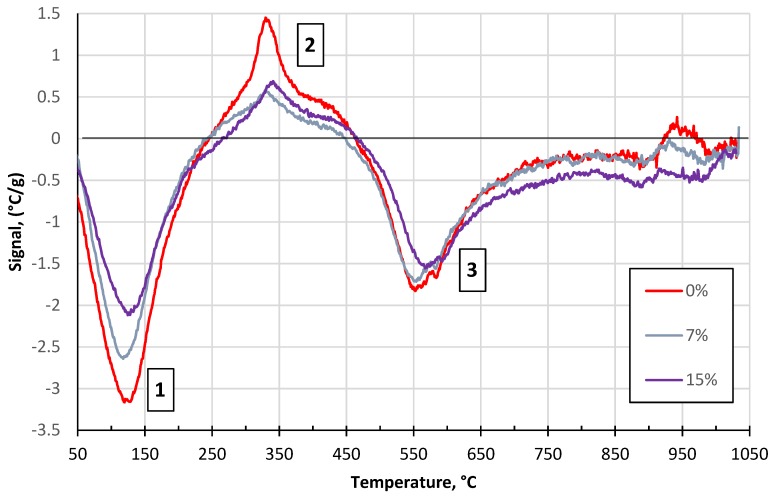
Differential thermal analysis (DTA) results of the tested clay-glass ceramic materials in the temperature range from 20 up to 1100 °C. The content of glass in wt% is in the legend.

**Figure 3:**
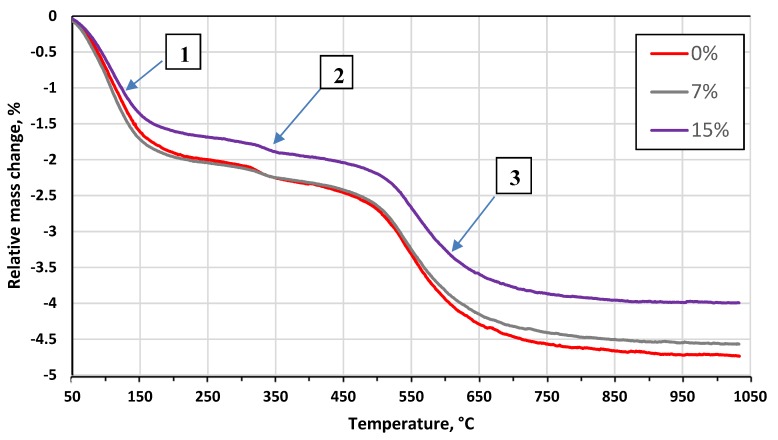
Thermogravimetric (TG) analysis results of the tested clay-glass ceramic materials in temperature range from 20 up to 1050 °C. Detected events (1, 2, and 3) are indicated. The content of glass in wt% is in the legend.

The results of the DTA and TG exhibited two endothermic and one exothermic reaction in all cases, as shown in Figure 2 and Figure 3, respectively.

**Figure 4:**
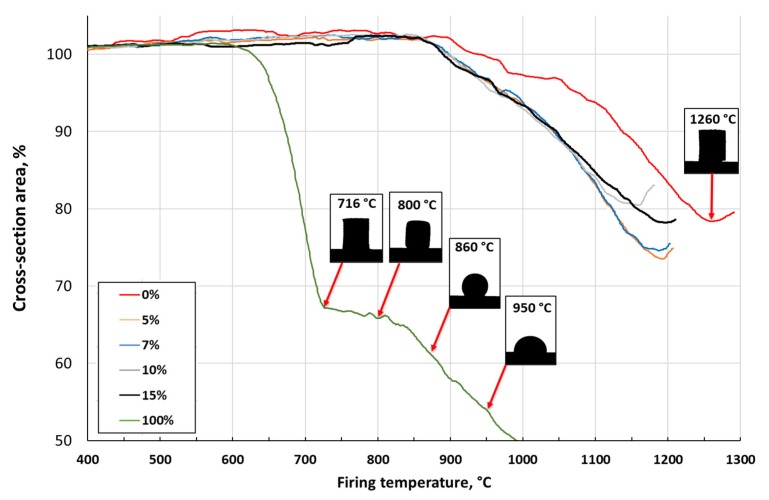
Dilatometric curves of the tested clay-glass ceramic materials in temperature range from 20 up to 1300 °C. Detected events are indicated. The content of glass in wt% is in the legend.

**Figure 5:**
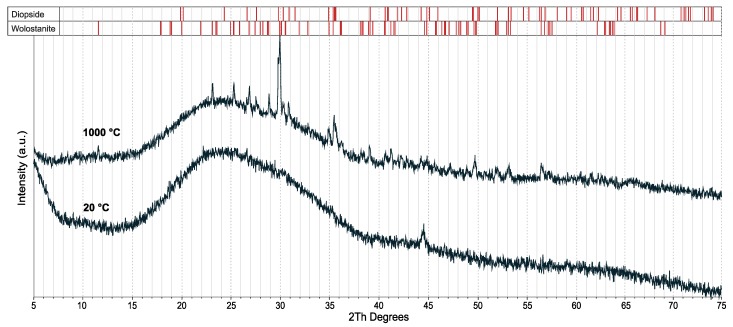
XRD pattern of the glass cullet (GC) (bottom curve) and GC treated at 1000 °C (upper curve).

**Figure 6:**
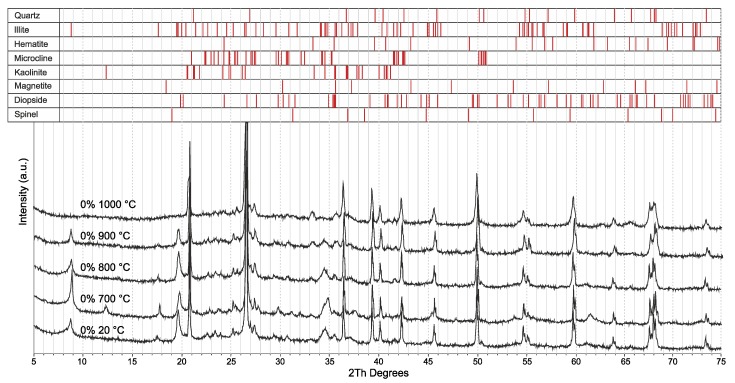
XRD patterns of Liepa clay fired at 700, 800, 900, and 1000 °C (from the bottom to top).

**Figure 7:**
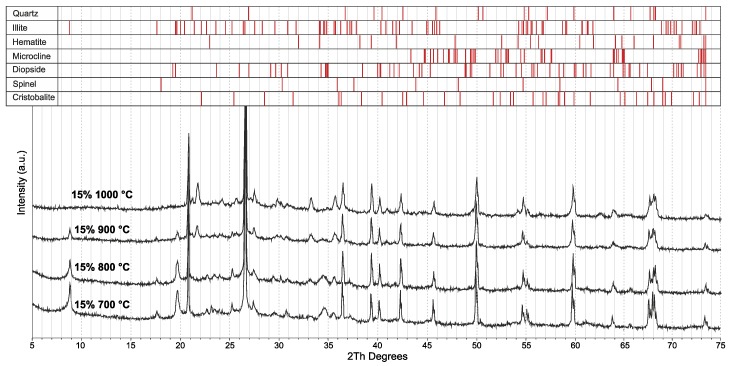
XRD patterns of Liepa clay with 15% of GC fired at 700, 800, 900, and 1000 °C (from bottom to top).

The first endotherm in the temperature range between 20 and 200 °C (see event N^o^ 1) is linked to a relative mass loss of 2% in the case of the pure clay (Figure 2 and Figure 3). This effect indicates the removal of the physically adsorbed water (and the rest of the water that was added during the preparation) and decomposition of crystal hydrates. Oxidation and decomposition of organic matter characterize the observed exothermic effect in the temperature range between 200 and 400 °C, as shown in Figure 2 and Figure 3 (see event N° 2). The second dominant endothermic peak is linked to the dehydroxylation of the clay mineral – illite, between 400 °C and 700 °C, accompanied with mass loss (see event N^o^ 3). This process causes a decrease in the sample’s mass as the constituent water evaporates. The decomposition of MgCO_3_ (700–800 °C) and CaCO_3_ (800–900 °C), and the reduction of Fe_2_O_3_ to FeO, exhibits low intensities, confirming the low concentrations of 1.32, 0.83, and 7.15 wt%, respectively [32], as shown in Figure 2 and Figure 3 (see event N^o^ 3). The intensity of the observed effects decreased with the concentration of the glass. The increase in glass concentration, up to 15 wt%, led to similar intensities in the DTA and TG plots, as compared to the previous mixture (5 wt%). The glass transition at 600–716 °C (Figure 4) not affecting the common DTA and TG patterns. At temperatures above 900 °C, the creation of new phases – spinel and cristobalite, was observed by the small exotherm effect at 900 °C (Figure 2). The formation of new phases in the glass and clay minerals reaction was the XRD pattern of the pure glass (Figure 5), where the formation of cristobalite during the thermal treatment was not observed. Additional evidence of the formation of new phases was also supported by XRD analysis (Figure 6 and Figure 7).

Dilatometric analysis of the clay showed the beginning of the sintering at 900 °C, as seen in Figure 4. The silhouette of the clay sample did not change its shape during heating up to 1260 °C. The transformation temperature of the glass occurred around 600 °C. The Littleton softening point of the tested glass occurred at 716 °C, at which the viscosity for glass is 6.6 Pa/s [25,33]. Densification of the tested glass ended at 900 °C with a subsequent liquid phase formation, which caused swelling at temperatures up to 1000 °C. Furthermore, the increase in temperature up to 1000 °C reduced the viscosity [33,34].

The addition of any amount (5–15 wt%) of GC decreased the onset of the clay’s sintering from 900 °C down to 860 °C. The course of the dilatometric curves did not differ significantly for the prepared samples (linear expansion up to the onset of the sintering, then shrinkage). Thus, the sintering was mainly governed by the clay in the samples. Once melted, the glass filled the pores in the ceramic body, thus leading to a more pronounced densification. This behavior highlights the role of the glass in the sample.

The phase composition of the studied samples below 900 °C showed a gradual increase in the amorphous content with glass content in the mixtures. Above 900 °C, the mineral illite underwent a structural collapse, and a glassy phase formed in accordance with the results reported for a clay with high illite content in [8,35,36,37]. Owing to this transition, the amorphous content increased further. After heating at 1000 °C (without glass addition), quartz (PDF#01-077-1060), muscovite (PDF#00-007-0025), illite (PDF#01-076-5970), diopside (PDF#01-071-3828), haematite (PDF#00-033-0664), and microcline (PDF#01-076-6582) were identified as mineral phases present in the sample (Figure 6 and Figure 7). The glass addition gave rise to a new mineral phase, as cristobalite (PDF# 00-027-0605) appeared in the fired samples. At temperatures above 960 °C, the silica tends to form cristobalite, which explains the increase in the cristobalite content with temperature [38]. On the other hand, the glass addition led to a decrease in the quartz content after the highest firing temperature. When comparing the samples CLWG 0% and CLWG 15%, it is evident that some mineral phases – notably, magnetite – vanished, but diopside formed at lower temperatures (at 800 °C) and in a higher amount, while only cristobalite appeared as a new mineral phase. The amounts of the crystalline phases and the total degree of crystallinity are represented in Table 2. The amount of each crystalline phase was calculated from only the crystalline parts (amorphous phase excluded). It can be clearly seen that the glass addition, rather than increasing the amorphous content, supports the crystallization at high temperatures. 

### 3.3. Effect of Sintering Temperature and Glass Concentration on the Physico-Mechanical Properties of the Clay Ceramics

**Figure 8:**
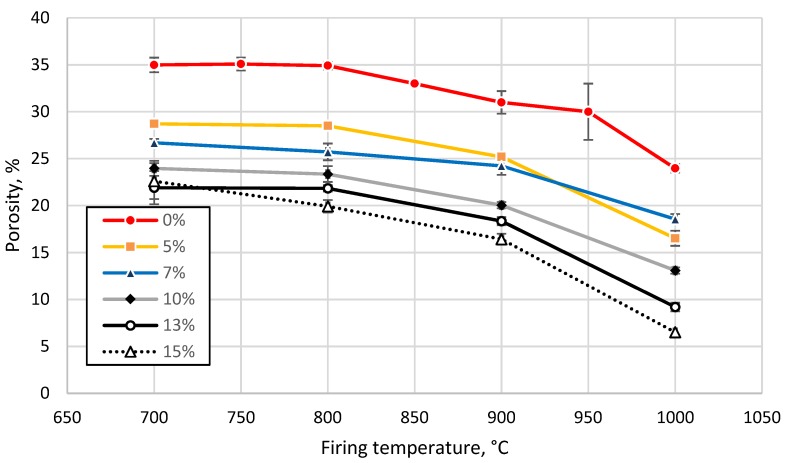
Apparent porosity of tested clay-glass ceramic materials after sintering at temperatures from 700 up to 1000 °C. The content of glass in wt% is in the legend.

**Figure 9:**
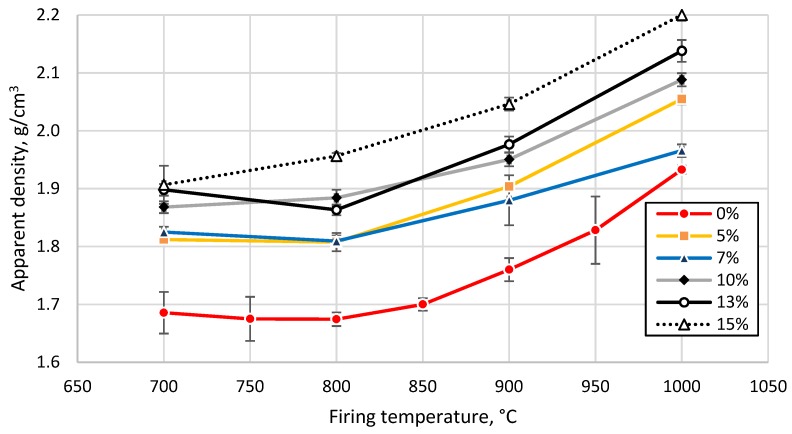
Apparent density of tested clay-glass ceramic materials after sintering at temperatures from 700 up to 1000 °C. The content of glass in wt% is in the legend.

**Figure 10:**
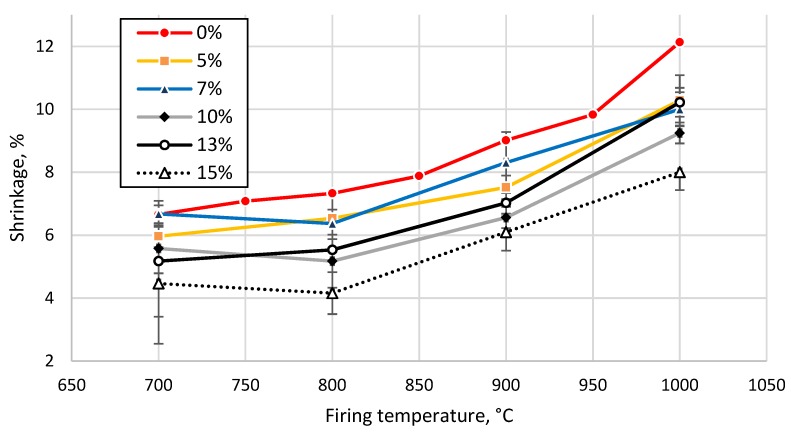
Shrinkage of tested clay-glass ceramic materials after sintering at temperatures from 700 up to 1000 °C. The content of glass in wt% is in the legend.

**Figure 11:**
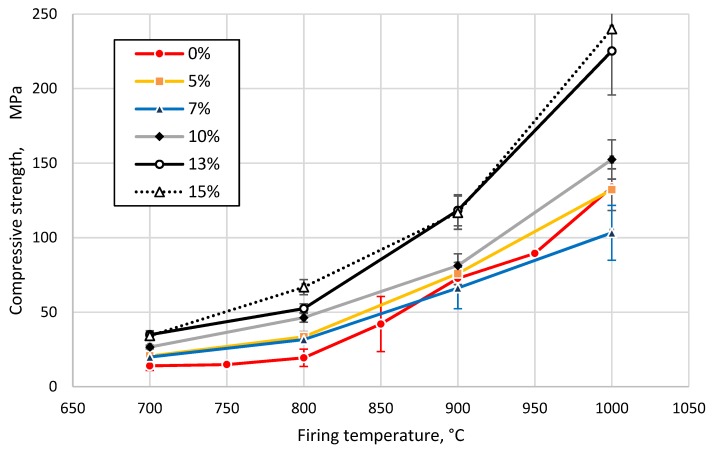
Compressive strength of tested clay-glass ceramic materials after sintering at temperatures from 700 up to 1000 °C. The content of glass in wt% is in the legend.

To obtain detailed information about the behavior (water uptake capacity, porosity, apparent density, etc.) of the prepared samples during the firing process, the samples were fired to different temperatures and subjected to mechanical tests. First, samples prepared from the clay without GC addition were prepared and fired at different temperatures from 700 to 1000 °C at 50 °C intervals. Secondly, samples prepared from the clay and GC were fired at different temperatures ranging from 700 to 1000 °C with 100 °C intervals. The trend of the curves exhibited a similar character; thus, a 100 °C temperature step can be considered short enough for a thorough description of the evolution of the physico-mechanical properties of the prepared samples (Figure 8, Figure 9, Figure 10 and Figure 11).

The water uptake capacity decreased from 21 to 13% for clay without GC addition, and from 11 to 3% for a GC loading of 15%, with an increase in the firing temperature from 700 to 1000 °C. Densification of the tested clay after sintering at temperatures above 850 °C was related to a decrease in the open porosity, as detected by the thermodilatometric analysis shown in Figure 4. The decrease in the open porosity, in turn, led to a decrease in the water uptake capacity. An increase in the sintering temperature above 850 °C led to a decrease in the apparent porosity from 35% (700 °C–800 °C) to 24% (1000 °C). The apparent density increased accordingly, from 1.68 to 1.93 g/cm^3^, as shown in Figure 8 and Figure 9, respectively.

The addition of the tested glass feedstock, up to 5 wt%, reduced the apparent porosity by 6%, after sintering at all the studied temperatures (Figure 8). An increase in glass concentration led to an increase in the apparent density by 2% (7 wt%, 1000 °C) and by 30% (15 wt%, 900 °C), as shown in Figure 9. The apparent porosity of all tested clay-glass mixtures decreased below 20% after sintering at 1000 °C. Shrinkage of the tested clay increased from 7% up to 12% with an increase in sintering temperature from 700 °C to 1000 °C. The addition of glass led to a decrease of up to 4% in the overall shrinkage of the ceramic body during the sintering of the sample with 15 wt% of GC at 1000 °C, as demonstrated in Figure 10 and confirmed by the interpretation of the endo- and exothermic processes detected in the DTA measurements (see Figure 2).

The compressive strength of all tested materials increased with the sintering temperature. However, its growth was more pronounced above 800 °C, as shown in Figure 11 and confirmed by TD, as demonstrated in Figure 4. The compressive strength increased in the case of samples with a glass content of more than 10 wt%, reaching 240 MPa (15 wt%, 1000 °C).

The added disintegrated glass acted as a degreaser in the plastic clay-water mixture, due to its almost non-plasticity, with the concomitant reduction of humidity inside the extruder and the consequent reduction in the drying shrinkage [39], as demonstrated in Figure 10. The presented effect implies a lower risk of drying caused by breakage and segregation (i.e., cracking) inside the glass containing clay. As may be seen from the Figure 11, the most significant increase in mechanical properties had specimens fired at 800 °C and above with 13 and 15 wt% GC loading. The compression strength increased on average by 250% at 800 °C, 60% at 900 °C, and 80% at 1000 °C in comparison to the clay without GC. At the same time, the apparent density did not increase much. These effects could be explained less by the appearance of new phases and crystallization, as reported by Bernardo et al. [40], and more by better clay-GC particle packaging in the mixture (according to Fuller’s curve [41]), where 13–15% of bigger GC particles were surrounded by much smaller clay particles and transitted to a low-viscosity state at temperatures above 716 °C (Littelton point, [25,33]) and over 860 °C; the “absorbed” nearest particles formed in this way vitrified agglomerates after the specimen cooled down.

The added GC acted as a fluxing agent during sintering of the dried clay-glass mixture. Once melted, the glass filled the pores of the ceramic material and contributed to its densification [42], as shown in Figure 9. The addition of the glass reduced the firing temperature (down to 860 °C) at which sintering of the tested clay occurred. 

**Figure 12:**
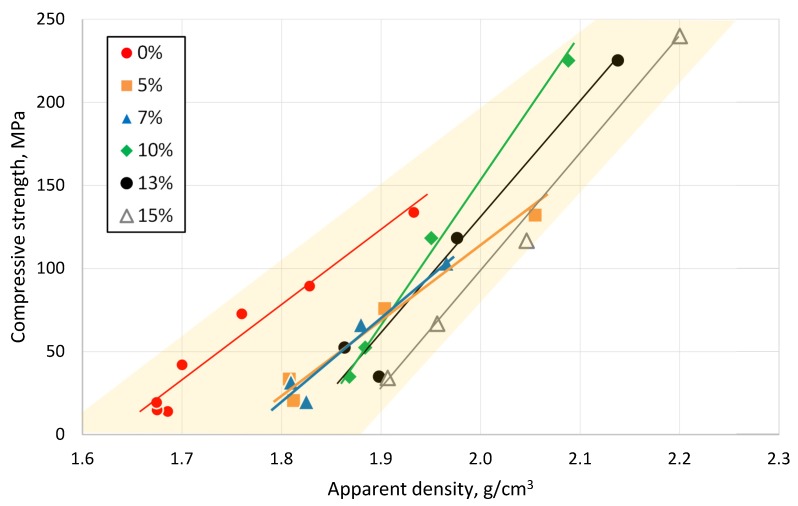
Dependence of compressive strength on apparent density of tested clay-glass ceramic materials after sintering at temperatures from 700 up to 1000 °C.

**Figure 13:**
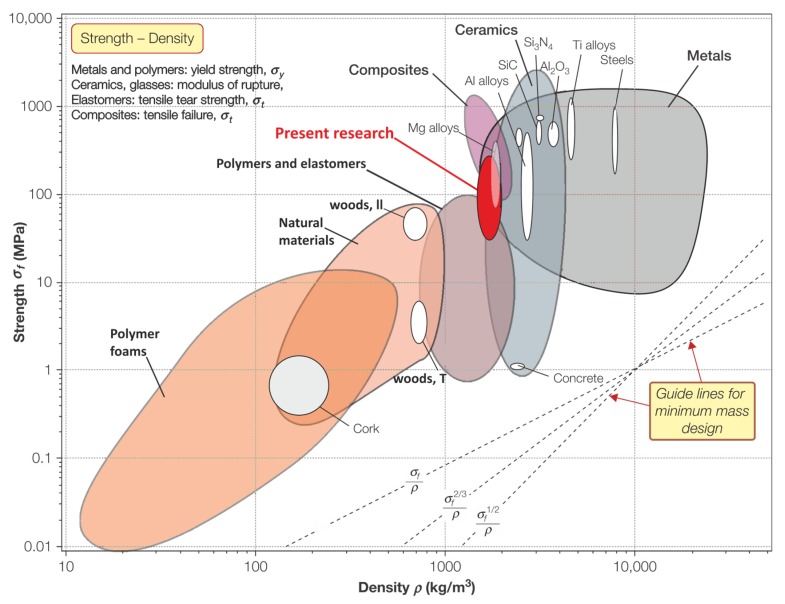
The compliance of produced clay-glass ceramic materials with typical materials demonstrated in the Ashby classification diagram (adopted with permission from [43]).

A higher clay-ceramic material density led to higher compressive strength values, as demonstrated in Figure 12. Many researchers have developed the classification of materials related to those between different selected parameters essential for specific applications. One of the most successful and practically applied approaches has been published by Ashby [43], plotting mechanical strength against the density of a material, as shown in Figure 13. Properties of the tested materials in the present work exhibit similar density values to the traditional ceramic products, and maintain a mechanical strength comparable to such high-strength polymers, like glass fiber-reinforced plastics (e.g., epoxy).

## 4. Conclusions

Selected clay from the Devonian Liepa clay deposit and municipal-waste green glass cullets were disintegrated twice with the dominant particle sizes down to *d*_50_clay_ = 0.035 and 0.0035 mm, and *d*_50_glass_ = 0.1 mm, respectively. Two cycles of clay and glass cullet disintegration with the selected disintegrator DSL = 175 required a total specific electrical energy of 24.48 kJ/kg. The addition of the disintegrated glass to the clay reduced the beginning of the clay sintering temperature from 900 °C to 860 °C due to the melting of the glass. The glass addition slightly decreased the typical clay endo- and exothermic DTA peak intensities in the temperature range from 20 °C up to 1000 °C due to the reduced concentration of clay minerals in the mixtures. Waste glass reduced the plasticity of the clay and reduced the risk of structural failure. By incorporating packaging glass waste into traditional ceramic products, not only economical but also environmental benefits can be gained. The use of waste materials decreased the production cost. In the present paper, it was proved that the addition of packaging waste glass brought about several benefits. First, compressive strength was increased. Secondly, water uptake was decreased. Thus, the incorporation of up to 15 wt% of glass waste to illitic clay products was proved to be beneficial from economic, environmental, and practical sides.

## Figures and Tables

**Table 1:** The chemical composition and LOI in wt% of the Liepa clay and bottle glass.

Material	SiO_2_	Al_2_O_3_	Fe_2_O_3_	CaO	MgO	Na_2_O	K_2_O	TiO_2_	LOI *
Clay	62.8 ± 0.5	15.4 ± 0.7	6.8 ± 0.2	0.7 ± 0.2	1.4 ± 0.2	0.1 ± 0.1	4.2 ± 0.1	1.9 ± 0.1	4.7 ± 0.1
Glass	70.20	2.10	0.10	9.50	–	16.60	–	–	1.50

* LOI - loss at ignition

**Table 2:** The phase composition of the fired clay and clay-glass ceramics.

Phase, %		CLWG 0%				CLWG 15%				Glass 100%	
	20 °C	700 °C	800 °C	900 °C	1000 °C	700 °C	800 °C	900 °C	1000 °C	20 °C	1000 °C
Quartz	40.8	60.9	60.3	71.0	72.6	57.7	58.7	69.1	67.9		
Illite	45.8		28.7	18.5		30.8	26.9	6.8			
Haematite	3.1	1.5	1.8	3.0	5.3	4.6	5.3	5.0	4.6		
Microcline	3.4	29.2	8.7	7.1	8.3	6.9	3.6	6.8	8.6		
Kaolinite	6.9										
Magnetite		0.51	0.49								
Diopside					1.7		5.5	4.8	5.3		60.4
Spinel					12.2			3.9	7.3		
Cristobalite								3.5	6.4		
Wollastonite											39.6
Amorphous phase, %	13.0	13.0	14.0	21.0	26.0	16.0	15.0	21.0	26.0	100	85.0

## References

[1] Eurostat Municipal waste landfilled, incinerated, recycled and composted in the EU-27, 1995 to 2015. http://ec.europa.eu/eurostat/statistics-explained/index.php/File:Municipal_waste_landfilled,_incinerated,_recycled_and_composted_in_the_EU-27,_1995_to_2015_update.png.

[2] Eurostats Treatment of waste by waste category, hazardousness and waste management operations. https://appsso.eurostat.ec.europa.eu/nui/show.do?dataset=env_wastrt&lang=en.

[3] Taveri G., Tousek J., Bernardo E., Toniolo N., Boccaccini A.R., Dlouhy I. (2017). Proving the role of boron in the structure of fly-ash/borosilicate glass based geopolymers. Mater. Lett..

[4] Hardy F. (1923). The physical significance of the shrinkage coefficient of clays and soils. J. Agric. Sci..

[5] Topçu İ.B., Canbaz M. (2004). Properties of concrete containing waste glass. Cem. Concr. Res..

[6] Rugele K., Lehmhus D., Hussainova I., Peculevica J., Lisnanskis M., Shishkin A. (2017). Effect of Fly-Ash Cenospheres on Properties of Clay-Ceramic Syntactic Foams. Materials.

[7] Švinka R., Švinka V. (1997). Silikātu materiālu ķīmija un tehnoloģija.

[8] Csáki Š., Štubňa I., Dobroň P., Minárik P., Záleská M., Václavů T., Vozár L. (2017). Influence of mechanical treatment on thermophysical processes in illitic clay during firing. Appl. Clay Sci..

[9] Csáki Š., Štubňa I., Trnovcová V., Ondruška J., Vozár L., Dobroň P. (2017). Evolution of AC conductivity of wet illitic clay during drying. IOP Conf. Ser. Mater. Sci. Eng..

[10] Silva R.V., de Brito J., Lye C.Q., Dhir R.K. (2018). The role of glass waste in the production of ceramic-based products and other applications: A review. J. Clean. Prod..

[11] Djangang C.N., Kamseu E., Elimbi A., Lecomte G.L., Blanchart P. (2014). Net-Shape Clay Ceramics with Glass Waste Additive. Mater. Sci. Appl..

[12] Húlan T., Kaljuvee T., Štubňa I., Trník A. (2016). Investigation of elastic and inelastic properties of Estonian clay from a locality in Kunda during thermal treatment. J. Therm. Anal. Calorim..

[13] Abuh M.A., Agulanna C.A., Chimezie P.E., Bethel-Wali J.U. (2019). Implications and characterization of waste glass cullet – kaolinite clay ceramics. J. Appl. Sci. Environ. Manag..

[14] Costa F.B., Teixeira S.R., Souza A.E., Santos G.T.A. (2009). Recycling of glass cullet as aggregate for clays used to produce roof tiles. Rev. Mater..

[15] Ondruška J., Csáki Š., Štubňa I. (2019). Influence of waste glass addition on thermal properties of kaolin and illite. AIP Conf. Proc..

[16] Goel G., Kalamdhad A.S., Agrawal A. (2018). Parameter optimisation for producing fired bricks using organic solid wastes. J. Clean. Prod..

[17] Goel G., Kalamdhad A.S. (2018). A practical proposal for utilisation of water hyacinth: Recycling in fired bricks. J. Clean. Prod..

[18] Goel G., Kalamdhad A.S. (2017). Degraded municipal solid waste as partial substitute for manufacturing fired bricks. Constr. Build. Mater..

[19] Goel G., Kalamdhad A.S. (2017). An investigation on use of paper mill sludge in brick manufacturing. Constr. Build. Mater..

[20] Phonphuak N., Kanyakam S., Chindaprasirt P. (2016). Utilization of waste glass to enhance physical–mechanical properties of fired clay brick. J. Clean. Prod..

[21] Bernardo E. (2007). Micro- and macro-cellular sintered glass-ceramics from wastes. J. Eur. Ceram. Soc..

[22] Jimenez-Millan J., Abad I., Jimenez-Espinosa R., Yebra-Rodriguez A. (2018). Assessment of solar panel waste glass in the manufacture of sepiolite based clay bricks. Mater. Lett..

[23] Andreola F., Barbieri L., Lancellotti I., Leonelli C., Manfredini T. (2016). Recycling of industrial wastes in ceramic manufacturing: State of art and glass case studies. Ceram. Int..

[24] Shishkin A., Mironov V., Zemchenkov V., Antonov M., Hussainova I. (2016). Hybrid Syntactic Foams of Metal –Fly Ash Cenosphere–Clay. Key Eng. Mater..

[25] Souza M.T., Maia B.G.O., Teixeira L.B., de Oliveira K.G., Teixeira A.H.B., Novaes de Oliveira A.P. (2017). Glass foams produced from glass bottles and eggshell wastes. Process Saf. Environ. Prot..

[26] Goljandin D., Sarjas H., Kulu P., Käerdi H., Mikli V. (2012). Metal-Matrix Hardmetal/Cermet Reinforced Composite Powders for Thermal Spray. Mater. Sci..

[27] Bumanis G., Goljandin D., Bajare D. (2016). The Properties of Mineral Additives Obtained by Collision Milling in Disintegrator. Key Eng. Mater..

[28] Zimakov S., Goljandin D., Peetsalu P., Kulu P. (2007). Metallic powders produced by the disintegrator technology. Int. J. Mater. Prod. Technol..

[29] Mironov V., Indriksone E., Goljandin D., Shishkin A. (2014). Obtaining of SiC Fine Powder from the Used Heating Elements by Milling and Grinding by High-Energy Disintegrator. Proceedings of the 23rd International Baltic Conference on Materials Engineering.

[30] Shishkin A., Mironov V., Goljandin D., Lapkovsky V. (2012). Recycling of Al-W-B Composite Material. Key Eng. Mater..

[31] (2002). Keramikas flīzes -3. daļa. Ūdens absorbcijas, šķietamās porainības, šķietamā un patiesā blīvuma noteikšana LVS. LVS EN 10545-3:2002.

[32] Muter O., Potapova K., Nikolajeva V., Petrina Z., Griba T., Patmalnieks A., Svinka R., Svinka V. (2012). Comparative study on bacteria colonization onto ceramic beads originated from two Devonian clay deposits in Latvia. Mater. Sci. Appl. Chem..

[33] Fluegel A. (2007). Glass viscosity calculation based on a global statistical modelling approach. Glas. Technol. Eur. J. Glas. Sci. Technol. Part A.

[34] Felisberto R., Santos M.C., Arcaro S., Basegio T.M., Bergmann C.P. (2018). Assessment of environmental compatibility of glass–ceramic materials obtained from galvanic sludge and soda–lime glass residue. Process Saf. Environ. Prot..

[35] Ondruška J., Trnovcová V., Štubňa I., Csáki Š., Vozár L. (2019). Influence of texture on DC conductivity and dimensional changes of kaolin and illitic clay. Ceram. Int..

[36] Csáki Š., Ondruška J., Trnovcová V., Štubňa I., Dobroň P., Vozár L. (2018). Temperature dependence of the AC conductivity of illitic clay. Appl. Clay Sci..

[37] Ondro T., Húlan T., Al-Shantir O., Csáki Š., Václavů T., Trník A. (2019). Kinetic analysis of the formation of high-temperature phases in an illite-based ceramic body using thermodilatometry. J. Therm. Anal. Calorim..

[38] Ibrahim D.M., Helmy M. (1981). Crystallite growth of rice husk ash silica. Thermochim. Acta.

[39] Loryuenyong V., Panyachai T., Kaewsimork K., Siritai C. (2009). Effects of recycled glass substitution on the physical and mechanical properties of clay bricks. Waste Manag..

[40] Bernardo E., Bonomo E., Dattoli A. (2010). Optimisation of sintered glass–ceramics from an industrial waste glass. Ceram. Int..

[41] Zhang Z., Song X., Liu Y., Wu D., Song C. (2017). Three-dimensional mesoscale modelling of concrete composites by using random walking algorithm. Compos. Sci. Technol..

[42] Lin K.L., Lee T.C., Hwang C.L. (2015). Effects of sintering temperature on the characteristics of solar panel waste glass in the production of ceramic tiles. J. Mater. Cycles Waste Manag..

[43] Ashby M.F. (2010). Materials Selection in Mechanical Design.

